# Expression, Tissue Distribution and Function of miR-21 in Esophageal Squamous Cell Carcinoma

**DOI:** 10.1371/journal.pone.0073009

**Published:** 2013-09-10

**Authors:** Nazila Nouraee, Katrien Van Roosbroeck, Mohammad Vasei, Shahriar Semnani, Nader Mansour Samaei, Farshad Naghshvar, Abbas Ali Omidi, George A. Calin, Seyed Javad Mowla

**Affiliations:** 1 Department of Molecular Genetics, Faculty of Biological Sciences, Tarbiat Modares University, Tehran, Iran; 2 Department of Experimental Therapeutics, MD Anderson Cancer Center, University of Texas, Houston, Texas, United States of America; 3 Pathology Laboratory, Shariati Hospital, Tehran University of Medical Sciences, Tehran, Iran; 4 Golestan Research Center of Gastroenterology and Hepatology, Golestan University of Medical Sciences, Gorgan, Iran; 5 Human Genetics Department, Golestan University of Medical Sciences, Gorgan, Iran; 6 Department of Pathology, Mazandaran University of Medical Sciences, Sari, Iran; 7 Department of Pathology, Mashhad University of Medical Sciences, Mashhad, Iran; Vanderbilt University, United States of America

## Abstract

**Objective:**

MiR-21 is an *oncomir* expressed by malignant cells and/or tumor microenvironment components. In this study we focused on understanding the effects of stromal miR-21 on esophageal malignant cells.

**Design:**

MiR-21 expression was evaluated in formalin-fixed paraffin-embedded samples from patients with esophageal squamous-cell carcinoma (SCC) by quantitative RT-PCR. MiR-21 tissue distribution was visualized with *in situ* hybridization. A co-culture system of normal fibroblasts and esophageal cancer cells was used to determine the effects of fibroblasts on miR-21 expression levels, and on SCC cell migration and invasion.

**Results:**

MiR-21 was overexpressed in SCCs, when compared to the adjacent non-tumor tissues (P = 0.0007), and was mainly localized in the cytoplasm of stromal cells adjacent to malignant cells. Accordingly, miR-21 expression was increased in tumors with high versus low stromal content (P = 0.04). When co-cultured with normal fibroblasts, miR-21 expression was elevated in SCC cells (KYSE-30), while its expression was restricted to fibroblasts when co-cultured with adenocarcinoma cells (OE-33 and FLO-1). MiR-21 was detected in conditioned media of cancer cell lines, illustrating the release of this miRNA into the environment. Co-culturing with normal fibroblasts or addition of fibroblast conditioned media caused a significant increase in cell migration and invasion potency of KYSE-30 cells (P<0.0001). In addition, co-culturing cancer cells with fibroblasts and expression of miR-21 induced the expression of the cancer associated fibroblast (CAF) marker S100A4.

**Conclusions:**

MiR-21 expression is mostly confined to the SCC stroma and its release from fibroblasts influences the migration and invasion capacity of SCC cells. Moreover, miR-21 may be an important factor in “activating” fibroblasts to CAFs. These findings provide new insights into the role of CAFs and the extracellular matrix in tumor microenvironment formation and in tumor cell maintenance, and suggest miR-21 may contribute to cellular crosstalk in the tumor microenvironment.

## Introduction

MicroRNAs (miRNAs) are short (∼22 nucleotides), endogenous non-coding RNAs, which act as post-transcriptional modulators of a variety of cellular processes including development, proliferation, differentiation and apoptosis [1]–[3]. Although microRNAs were initially found to inhibit translation or degrade their mRNA targets by imperfect or perfect complementary binding, new publications have assigned other regulatory roles for miRNAs, including promoter companionship [4]. Alterations in the expression of miRNAs are associated with a variety of diseases including cancer, where they show tumor-specific expression signatures. Therefore, targeting miRNAs might hold great diagnostic and therapeutic promise [5]–[7].

Increasing evidence implicates miR-21 as an “oncomir” in tumorigenesis, where it is found to be upregulated in the majority of analyzed cancers, including glioblastoma, colorectal, breast and pancreatic cancer [8]–[13]. By regulating different targets, miR-21 is involved in cellular proliferation, evasion of apoptosis, epithelial to mesenchymal transition (EMT), and invasion [8], [14]–[16].

At the cellular level, the majority of studies focused on miR-21 overexpression in cancer, where, according to its oncogenic role in tumorigenesis, the highest miR-21 expression levels are expected in tumor cells [17]–[18]. However, in breast and colon cancer miR-21 has also been localized to cancer associated fibroblast-like cells (CAFs) [19]–[21]. These fibroblasts facilitate communication between the tumor cell and the tumor microenvironment, and thus support tumor progression, angiogenesis and metastasis. These findings point to a dynamic role of miR-21 in malignant behavior through stimulation of cancer cell proliferation and extracellular matrix (ECM) remodeling [19]–[21]. However, the precise role of miR-21 at the tissue level still needs to be elucidated. Among the well characterized molecular targets of miR-21 are tropomyosin 1 (*TPM1*) [22], tissue inhibitor of metalloproteinase 3 (*TIMP3*) [23]–[24], and components of the transforming growth factor beta (*TGFβ*) pathway [25]. TGFβ can induce apoptosis or proliferation, depending on the cellular context and the specific state of the cells. In addition, miR-21 can increase TGFβ signaling through targeting *SMAD* [26]–[28].

Esophageal squamous cell carcinoma (ESCC) is an aggressive type of epithelial cancer that is characterized by scarce overall survival and a low rate of response to (neo-) adjuvant therapy [29]. Hence, there is a great need for a multimodal treatment. In recent years, several molecular markers have been introduced as predictive and prognostic targets in patients with esophageal cancer [30].

Iran, and the Golestan province in particular, has one of the highest rates of esophageal cancer (EC) in the world [31]. Therefore, the aim of the current study was to analyze the expression of miR-21 in SCC samples of Iranian patients and to explore whether stromal-expressed miR-21 has an influence on the behavior of the malignant cells.

## Materials and Methods

### Clinical sample collection

A total of 42 formalin-fixed paraffin-embedded (FFPE) tissue samples of patients with esophageal SCC were collected from the archive of the Namazi hospital (Shiraz University of Medical Sciences, Iran). Hematoxylin and eosin (H&E) stained histopathological sections of each sample were further studied by an expert pathologist (MV) to delineate the tumor/non-tumor areas as well as the histopathological criteria of each sample. The pathological characteristics of the SCC patient samples are summarized in Table 1. The paired tumor and non-tumor areas of each FFPE block were carefully macro-dissected and transferred to an RNase-free microcentrifuge tube for RNA extraction.

**Table 1:** Histopathological criteria of the analyzed SCC patients.

	Differentiation level			Stroma level		Inflammation level	
Patient samples	Poor (high grade)	Moderate (intermediate grade)	Well (low grade)	Low	High	Low	High
Number	12	4	26	11	31	26	16
Percentage	28.57%	9.52%	60.9%	26.19%	73.81%	61.9%	38.1%

### Ethics statement

This study was reviewed and approved by the Ethical Committee of Tarbiat Modares University. All samples were collected according to the institutional policies. We used archival FFPE samples that were collected 10–20 years ago, and most patients deceased so no written consent could be obtained. Also, as the Guidelines for Record and Specimen Retention suggest a retention period for paraffin blocks and slides of 10 years [32], the archived FFPE samples from this study could be studied without any ethical concern. However, all patients from this study provided verbal informed consent at the time of admission to the hospital and the verbal consent procedure was reviewed and approved by the Ethical Committee of Tarbiat Modares University.

### RNA extraction from esophageal FFPE specimens

First, samples were deparaffinized with xylol and digested with Proteinase K solution (Fermentas, Lithuania). Several factors, including the proteinase K/buffer composition, temperature and digestion time, were altered to optimize the protein digestion procedure. The following protocol was then used: incubation in PK buffer (1 mM EDTA, 1 mM NaCl, 5 mM Tris-HCl, pH 7.4) supplemented with 15 μg/ml of proteinase K for 3 hours at 54°C. RNA was then extracted from deparaffinized tissue with TRIzol reagent (Invitrogen, USA) according to the manufacturer's instructions.

### MiR-21 quantification in FFPE samples by quantitative RT-PCR

After treatment with DNase I (Fermentas, Lithuania; manufacturer' s recommendations), 100 ng of total RNA was subjected to qRT-PCR, using a two-step protocol of universal cDNA synthesis and SYBR green master mix kits, along with specific locked nucleic acid (LNA) PCR primer sets (Exiqon, Denmark) on an ABI 7500 real-time PCR machine. To evaluate the possibility of contamination by any RT-PCR inhibitors, an RNA spike-in (UniSp6; Exiqon, Denmark) was added to the samples prior to cDNA synthesis (10^8^ copies per 20 ng RNA) and qRT-PCR was performed with spike-in PCR primer sets (Exiqon, Denmark) as well.

For each sample a no-reverse transcription (no-RT) control was used to detect any potential non-specific amplification of genomic DNA. 5S rRNA and U6 snRNA were used as internal controls for data normalization in FFPE and cell culture samples, respectively.

### *In situ* hybridization on FFPE samples of esophageal SCC

Deparaffinization by xylol and Proteinase K digestion (Fermentas, Lithuania) were performed as described in a previous section of the Experimental Procedures. Slides were then incubated with 5′ digoxigenin (DIG)-labeled miRCURY LNA microRNA detection probes (Exiqon, Denmark), which were diluted to 50 nM in hybridization buffer (50% Formamide, 5X SSC, 0.1% Tween-20, 9.2 mM citric acid, 50 μg/mL heparin, 500 μg/mL yeast RNA) for 1h in a ThermoBrite hybridizer (Fisher Scientific, USA). LNA antisense oligonucleotides were used to increase the oligo-miRNA binding affinity. The probe sequences were the following:

hsa-miR-21 probe: 5′-TCAACATCAGTCTGATAAGCTA-3′hsa/mmu/rno-U6 snRNA probe: 5′-CACGAATTTGCGTGTCATCCTT-3′.

A stringency wash was performed in descending serial dilutions of standard sodium citrate (SSC) buffer. After blocking unspecific binding of the antibody with sheep serum (Sigma, USA), immunological detection with alkaline phosphatase (AP)-conjugated sheep anti-DIG antibody (Roche, Germany) was carried out overnight at 4°C. A light-sensitive color reaction with 4-nitroblue tetrazolium and 5-bromo-4-chloro-3-indolyl phosphate (NBT/BCIP) ready-to-use tablets (Roche, Germany) was performed for 3 hours at 30°C in a humidified chamber.

### Cell culture conditions and cell lines

The KYSE-30 esophageal SCC cell line was obtained from the National Cell Bank of Iran, and cultured in RPMI 1640 (Gibco, USA) and Ham' s F12 (Invitrogen, USA) (1∶1) supplemented with 10% fetal bovine serum (FBS) (Sigma, USA). The esophageal adenocarcinoma cell lines OE-33 and FLO-1 originated from the American Type Culture Collection (ATCC) and were kindly provided by Dr. Dipen Maru (Department of Pathology, The University of Texas MD Anderson Cancer Center, Houston, TX, USA). OE-33 and FLO-1 were cultured in RPMI 1640 and DMEM Dulbecco' s Modified Eagle Media (Gibco, USA) supplemented with 10% FBS, respectively. Human normal fibroblasts of gingiva (HGF-1) were obtained from the American Type Culture Collection (ATCC) and cultured in DMEM supplemented with 10% FBS.

The conditioned media from HGF-1 fibroblasts and the esophageal cancer cell lines, containing secreted growth factors, were collected at different time points, centrifuged for 5 min at 1500 rpm, equally mixed with fresh media and used for further experiments.

### MiR-21 quantification in cell lines

For microRNA quantification in cell lines, a miR-21 and U6 snRNA specific reverse transcription reaction with MultiScribe Reverse Transcriptase and microRNA specific primers (Applied Biosystems, USA) was performed on 50 ng of total RNA. Quantitative RT-PCR was carried out with a Taqman assay (primers and probes) (Applied Biosystems, USA) and SsoFast Probe Supermix (BioRad, USA) according to manufacturer's protocol.

### Co-culture of esophageal cancer cell lines with normal human gingival fibroblasts (HGF-1)

To evaluate the effect of normal fibroblasts on miR-21 expression levels, we separately juxtaposed all 3 esophageal SCC and adenocarcinoma cell lines with HGF-1 fibroblast cells in a Transwell system. In this system, HGF-1 cells were seeded in the wells of a 12-well plate, while esophageal cancer cells were seeded in the 0.4 μm pore-sized inserts (BD Biosciences, USA) that were placed in each well to avoid physical contact between the two cell types. Both fibroblasts and cancer cells were harvested after 1, 2 and 3 days of incubation. The time point day 1 after co-culture was considered as a reference for comparison.

To overcome the effects of the use of different media on cell stress in the co-culture experiments, OE-33 and KYSE-30 cells, normally cultured in RPMI 1640 supplemented with 10% FBS and in RPMI 1640/Ham' s F12 (1∶1) supplemented with 10% FBS, respectively, were adapted to DMEM medium supplemented with 10% FBS. In three consecutive passages, cells were grown in mixtures of RPMI 1640 and DMEM supplemented with 10% FBS in a 3∶1, 1∶1 and 1∶3 ratio, respectively. From the fourth passage onwards, cells were cultured in DMEM supplemented with 10% FBS.

### Evaluating fibroblastic markers and CAF markers in HGF-1 cells

PCR primers for the fibroblast markers *TGFβ1* (MIM: 190180), *FGF1* (MIM: 131220), *STAT3* (MIM: 102582), *STAG2* (MIM: 300826), *TIMP3* (MIM: 188826), *COL4A1* (MIM: 120130) and for the CAF markers *ACTA2* (MIM: 102620), *FAP* (MIM: 600403), *S100A4* (MIM: 114210) and *CSPG4* (MIM: 601172) were designed over exon boundaries in order to amplify the most common splicing variants of each gene without amplifying the genomic DNA. Primer sequences for the fibroblast markers can be found in Table S1, CAF marker primer sequences are available upon request. Each primer pair was validated and all PCR products were confirmed by Sanger sequencing (Figure S1; data not shown).

Gene expression quantification was performed with SuperScript III reverse transcriptase (Invitrogen, USA) for the reverse transcription reaction and iQ™ SYBR Green Supermix (Biorad, USA) for qRT-PCR. *β2M* was, among 8 common normalizers (*PGK1*, *HPRT1*, *GUSB*, *PPIA*, *RPLPO*, *TBP, β2M* and *β Actin*) and based on Cq values (Figure S2), selected as the best internal control for these experiments. Primer sequences of the normalizers are available upon request.

All of the fibroblastic markers were predicted by databases (TargetScan and/or DIANA-microT) to be potentially targeted by miR-21, and some of them have already been validated as miR-21 targets.

### RNA extraction and miR-21 quantification from conditioned medium

The conditioned media from KYSE-30 and HGF-1 cells was harvested after 1, 2 and 3 days, centrifuged for 5 minutes at 1500 rpm and kept at −20°C for further experiments. RNA was extracted from 300 μl of conditioned medium with the Total RNA Purification Kit (Norgen, Canada). A mixture of 25 fmol of the synthetic *C. elegans* microRNAs cel-miR-39 and cel-miR-54 (Ambion, USA) were spiked in all samples immediately after adding lysis buffer. 10 ng of each sample was used for qRT-PCR analysis in which the synthetic miRNAs (Applied Biosystems, USA) were used as internal controls. Data were normalized to the expression in the 6 hour old media from each cell line.

### Migration assay

To determine the effects of normal fibroblasts on KYSE-30 migration, 7.5×10^4^ fibroblasts were seeded in 24-well plates. The next day, 7.5×10^4^ KYSE-30 cells were seeded in the upper chamber of Transwells with 8 μm pore size, which were uniformly coated with 0.1% gelatin (BD Biosciences, USA) and placed in the wells in which the fibroblasts were growing. KYSE-30 cells that were not co-cultured with fibroblasts were used as controls. After 16 hours of incubation, the membranes were fixed and stained using the Hema3 manual staining system (Fisher Scientific, USA). With a cotton swap, the cells in the upper surface of each membrane were removed and the cells on the bottom surface were counted under the microscope.

### Invasion assay

In this assay, the 8 μm pore-sized Transwell membranes (BD Biosciences, USA) were coated evenly with a matrix containing type IV collagen, human laminin and gelatin (Sigma, USA). 6.25×10^4^ cells were seeded in the upper chamber and co-cultured with previously seeded HGF-1 cells. The cells were fixed and stained after 20 hours of incubation with the aforementioned protocol for migration.

### Statistical analyses

For qRT-PCR analysis, at least 3 experiments were performed in triplicate and statistical analysis was carried out on ΔCq data. The reaction efficiencies for miRNA expression were determined with the LinRegPCR software (Amsterdam, The Netherlands, Version 12.12) (Table S2). Group-wise comparison between tumors and their non-tumor counterparts as well as statistical analysis of relative expression were performed with the “Relative Expression Software Tool” (REST) (Qiagen, Germany, Version 2.0.13). We used Microsoft Excel to analyze miR-21 expression levels in different cell culture experiments. The statistical difference between groups was determined by unpaired *t* test or unpaired *t* test with Welch's correction (when the data had significant unequal variances) and *P* values of less than 0.05 were considered as statistically significant. Data represent the mean +/− standard deviation (SD) of at least three independent experiments performed in duplicates or triplicates.

## Results

### MiR-21 is upregulated in esophageal tumor tissues

**Figure 1:**
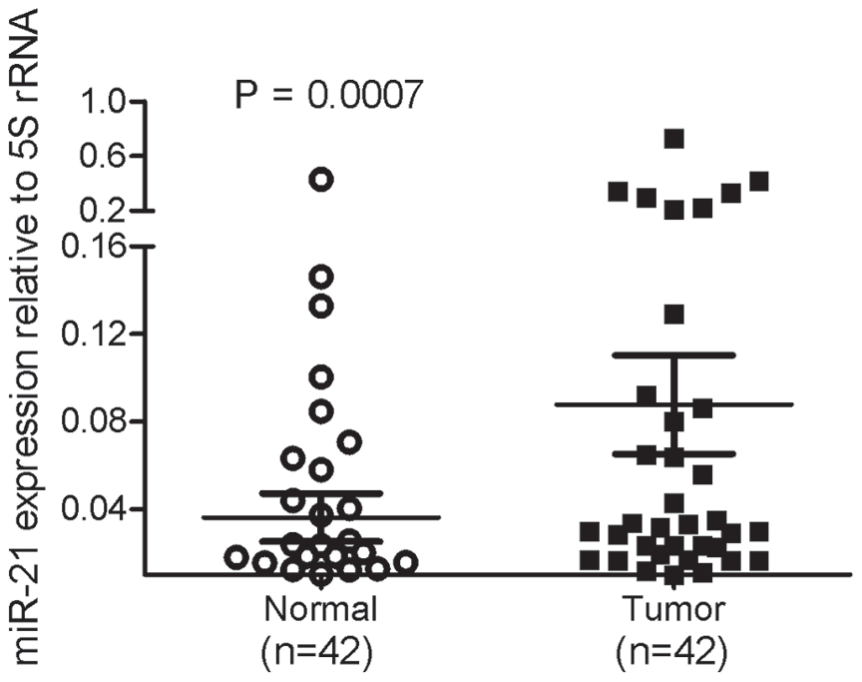
Differential expression of miR-21 in 42 FFPE tumor samples in comparison with adjacent non-tumoral tissue. Quantitative RT-PCR analysis on FFPE samples of esophageal SCC patients shows higher levels of miR-21 in cancerous tissue as compared to the adjacent non-cancerous counterpart (P = 0.0007). MicroRNA levels are normalized to 5S rRNA. Values are presented as means ± standard deviation. The P value was determined with a 2-tailed Student' s t-test.

Quantitative RT-PCR analysis on total RNA demonstrated a significant overexpression of miR-21 in esophageal SCC samples, in comparison to the adjacent, histologically normal tissues of the same patients (P = 0.007, **Figure 1**). We also compared the miR-21 expression levels in low- vs. high-grade tumors and found that miR-21 expression levels did not discriminate between well differentiated (considered as low-grade) and poorly differentiated (high-grade) tumors (P>0.05) (data not shown), suggesting that miR-21 overexpression is an early event in the tumorigenesis of esophageal tissue.

### MiR-21 is mainly localized in the cancer associated fibroblasts

**Figure 2:**
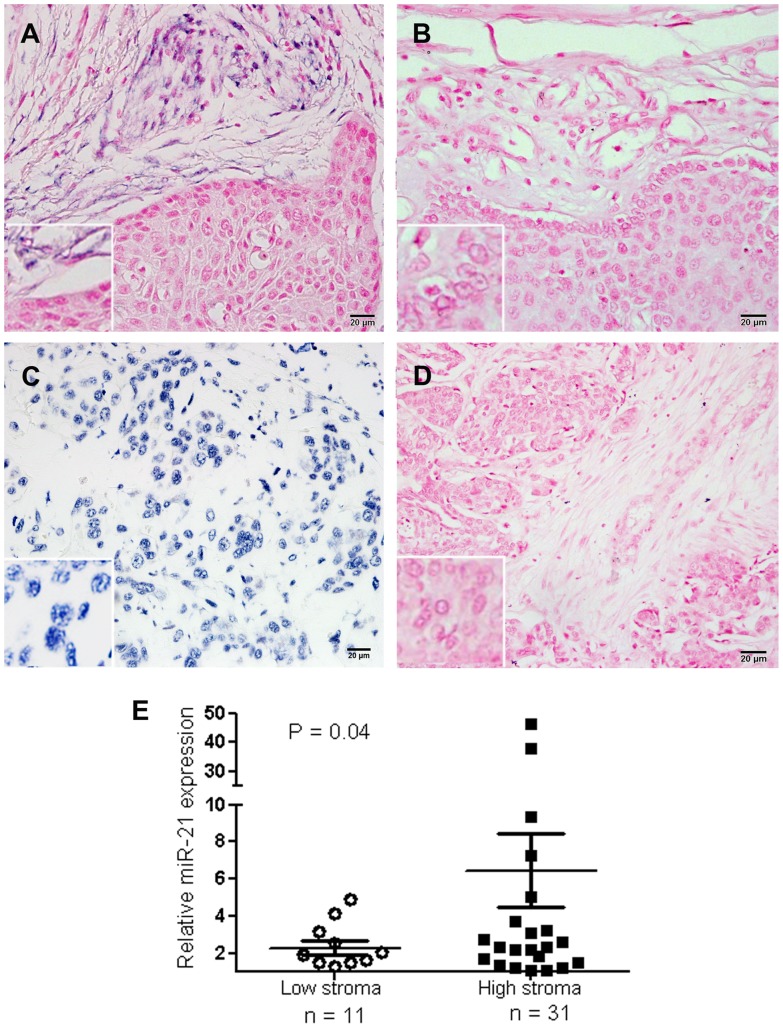
MiR-21 expression is mainly confined to the tumor stroma. A) *In situ* hybridization in FFPE samples of esophageal cancer localized miR-21 expression (blue signals) in cancer associated fibroblasts of the tumor stroma, but not in the tumor cells. Slides were counterstained with nuclear fast red. B) The adjacent normal squamous part on the same slide did not show miR-21 expression neither in the stroma nor in the squamous cells; C) Nuclear staining of U6 snRNA was used as an internal control; D) Negative control without probe. Bottom-left inserts show a 2 times bigger magnification of each image; E) Samples with high stromal component showed significantly higher levels of miR-21 expression than samples with a low stromal content. (P value  = 0.04 with unpaired T-test with Welch' s correction).

Using specific LNA-oligo probes against miR-21, we analyzed endogenous miR-21 expression in the FFPE sections prepared from either tumor or non-tumoral esophageal samples. *In situ* hybridization (ISH) demonstrated a primarily cytoplasmic signal of miR-21 in tumor regions; however, the signal was mainly found in cancer associated fibroblasts (CAFs) of the tumor stroma, with much weaker signals in the tumor cells (**Figure 2A**). The stroma of apparent normal squamous tissue showed no detectable signal (**Figure 2B**), suggesting a preferential upregulation of miR-21 in stromal fibroblasts adjacent to the tumor cells. This was observed in all analyzed samples (N = 6, **Figure S3**). The nuclear localization of U6 snRNA was used as an internal control (**Figure 2C**), and slides without probe treatment were used as negative controls (**Figure 2D**).

### MiR-21 expression levels are correlated with the stromal proportion of the tumors

Next, we re-examined miR-21 expression in the esophageal FFPE samples with different stromal ratios. Based on the stromal contents, determined by an expert pathologist for each sample, we categorized the samples into two groups: high stroma (more than 50%) vs. low stroma. The obtained qRT-PCR data demonstrate that miR-21 levels are significantly higher in tumors with high stroma (P = 0.04), as compared to those with low levels of stroma (**Figure 2E**).

### Induction of miR-21 expression in a co-culture assay of esophageal cancer cells and normal fibroblasts

**Figure 3:**
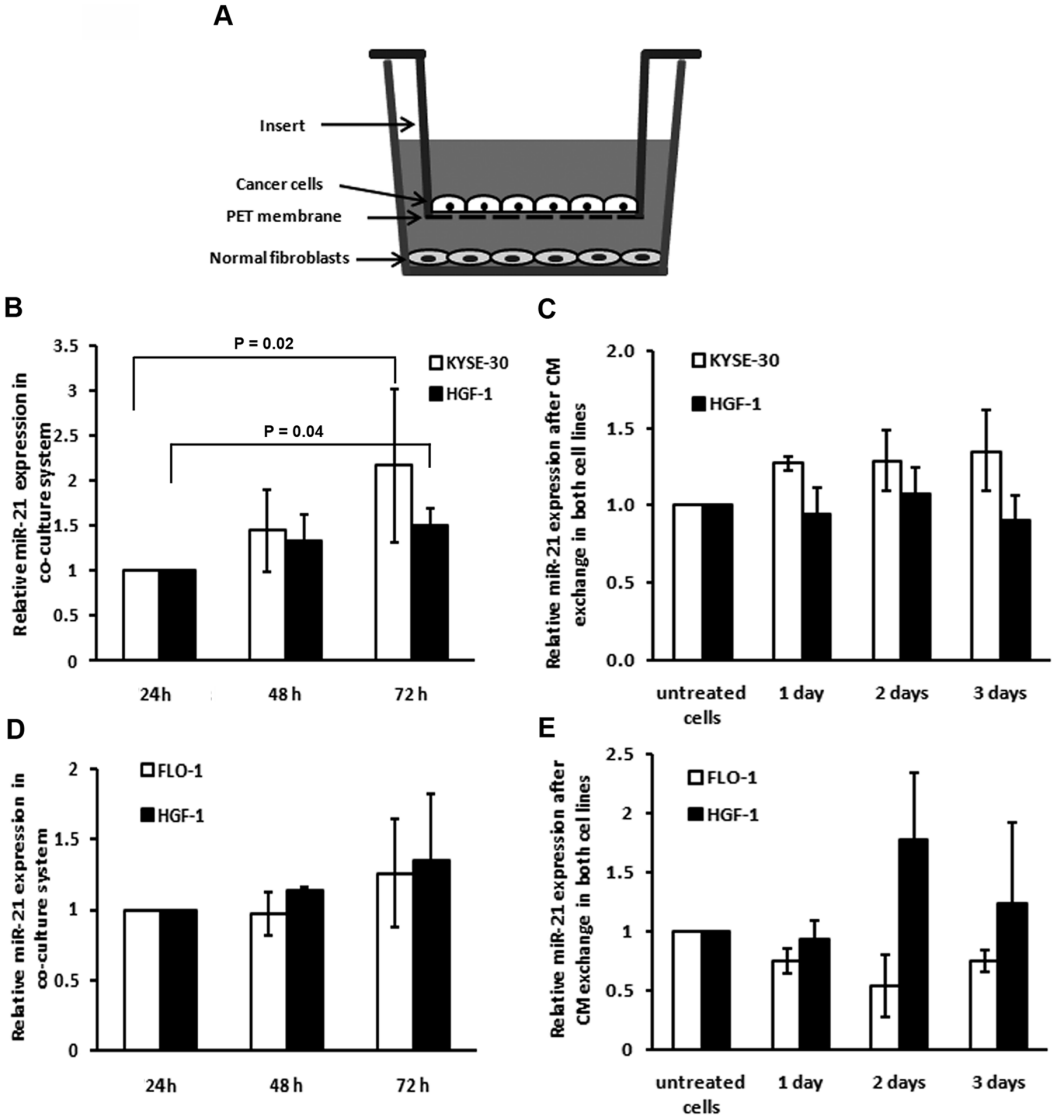
Effect of co-culturing normal fibroblasts with esophageal cancer cell lines on miR-21 expression levels. A) A schematic view of the co-culture system; B) MiR-21 expression in KYSE-30 cells and normal fibroblasts (HGF-1) after juxtaposition in a co-culture system; C) MiR-21 expression levels in KYSE-30 and HGF-1 cells after treatment with CM of HGF-1 and KYSE-30 cells, respectively; D) MiR-21 expression levels in FLO-1 adenocarcinoma and HGF-1 fibroblast cells after juxtaposition in a co-culture system; E) MiR-21 expression levels in FLO-1 and HGF-1 cells after treatment with CM of HGF-1 and FLO-1 cells, respectively. Data were normalized to expression after 24 h of co-culture. Each experiment was performed at least 2 times in triplicates.

To examine whether the tumor microenvironment has a role on miR-21 intra-tumor distribution, we used a co-culture system in which the fibroblasts that were cultured in a 12-well plate shared the media with the KYSE-30 cells that were grown on an insert located within the same well (**Figure 3A**). We then compared miR-21 expression in this co-culture system with miR-21 expression in fibroblasts grown without KYSE-30 cells. The fibroblasts showed a significant upregulation of miR-21 when grown in a co-culture system with KYSE-30 (P = 0.04; **Figure 3B** **, black bars**). Interestingly, we found a similar and significant effect of fibroblasts on the expression levels of miR-21 in KYSE-30 cells (P = 0.02; **Figure 3B** **, white bars**). This upregulating effect on miR-21 expression was even more obvious for HGF-1 fibroblast cells co-cultured with FLO-1 cells (**Figure 3D**). However, these differences were not statistically significant.

To understand the nature of this micro-environmental effect, we added conditioned media (CM) obtained from the normal fibroblasts to the esophageal SCC cell line KYSE-30 and to the adenocarcinoma cell line FLO-1. As shown in **Figure 3C**, the fibroblast conditioned media induces miR-21 expression in the KYSE-30 cells (white bars), with longer conditioning period leading to higher miR-21 expression. Moreover, treatment of HGF-1 cells with CM from FLO-1 induced higher expression levels of miR-21 in the HGF-1 fibroblasts (**Figure 3E** **, black bars**). The basic miR-21 expression levels were not significantly different in all 4 cell lines used in these experiments (**Figure S4**). Altogether, these results indicate that the effect of neighboring fibroblasts on the cancer cells might be different in different histological subtypes.

### Both cancer cells and normal fibroblasts can release miR-21 into the environment

Quantitative RT-PCR on RNA extracted from the conditioned media of fibroblast cultures showed an up to 8.4 fold overexpression of miR-21 after 3 days of incubation, relative to the media which was collected from the same cell passage after 6 hours of culturing (P = 0.02). In addition, we observed a 9.3 fold increase in miR-21 expression in the 3 day old conditioned media from KYSE-30 cells, relative to the media collected from the same cell passage after 6 hours, but this increase was not significant (Figure S5).

### Normal fibroblasts as well as conditioned media from normal fibroblasts can potentiate migration and invasion of neighboring cancer cells

**Figure 4:**
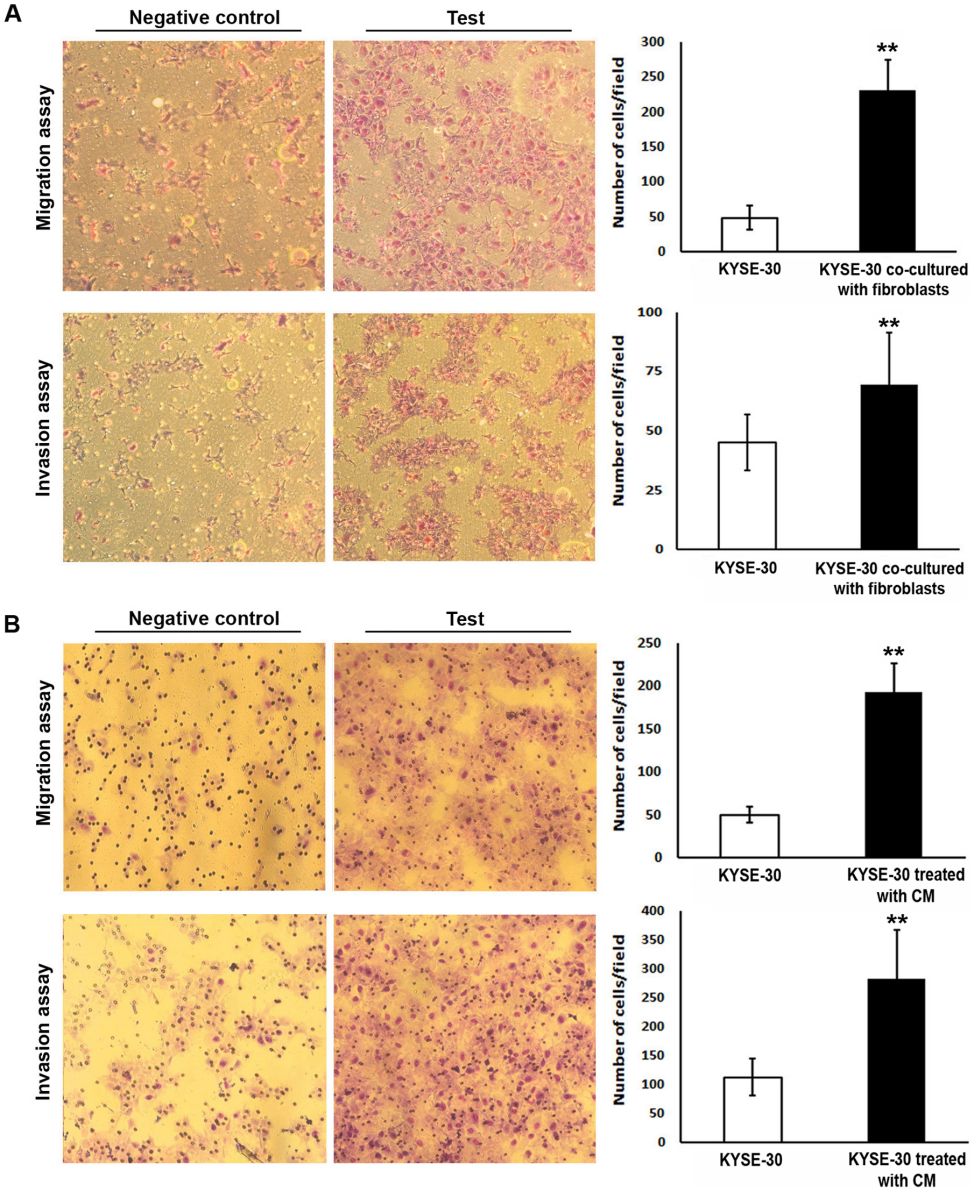
Cell migration and invasion properties of KYSE-30 cells are increased upon co-culture with HGF-1. A) KYSE-30 cells juxtaposed with HGF-1 fibroblasts showed a significantly higher potential to migrate (P<0.0001) or invade (P = 0.0001) through the coated 8.0 μm pore-sized membrane as compared to the KYSE-30 cells not grown co-culture with normal fibroblasts. Images of representative microscopic pictures are shown on the left; B) 3 day old conditioned media from HGF-1 fibroblasts could significantly induce migration and invasion of KYSE-30 cells (P<0.0001 for both experiments). Images of representative microscopic pictures are shown on the left.

While juxtaposed with normal fibroblasts, KYSE-30 cells show a significantly higher propensity to migrate and invade through the matrix, which simulates the ECM. KYSE-30 cells not juxtaposed with normal fibroblasts were used as negative controls (**Figure 4A**). Incubation of KYSE-30 cells with the conditioned media of HGF-1 fibroblast cells also results in significantly higher rates of migration (P<0.0001) and invasion (P<0.0001) of KYSE-30 cells through the matrix (**Figure 4B**). To investigate the role of induced miR-21 in the capacity of KYSE-30 cells to migrate and invade through the matrix, we inhibited miR-21 in HGF-1 prior to co-culturing with KYSE-30. We found that miR-21 was significantly downregulated at the end of the experiment (**Figure S6A–B**) and that KYSE-30 cell migration and invasion was reduced, although this reduction was not significant (**Figure S6C–D**). In addition, *TIMP3* and *COL4A1* seem to be increased in HGF-1 cells treated with miR-21 inhibitor and co-cultured with KYSE-30 (**Figure S6E–F**).

### The fibroblastic markers *TIMP3* and *COL4A1* showed decreased expression levels in the co-culture system

**Figure 5:**
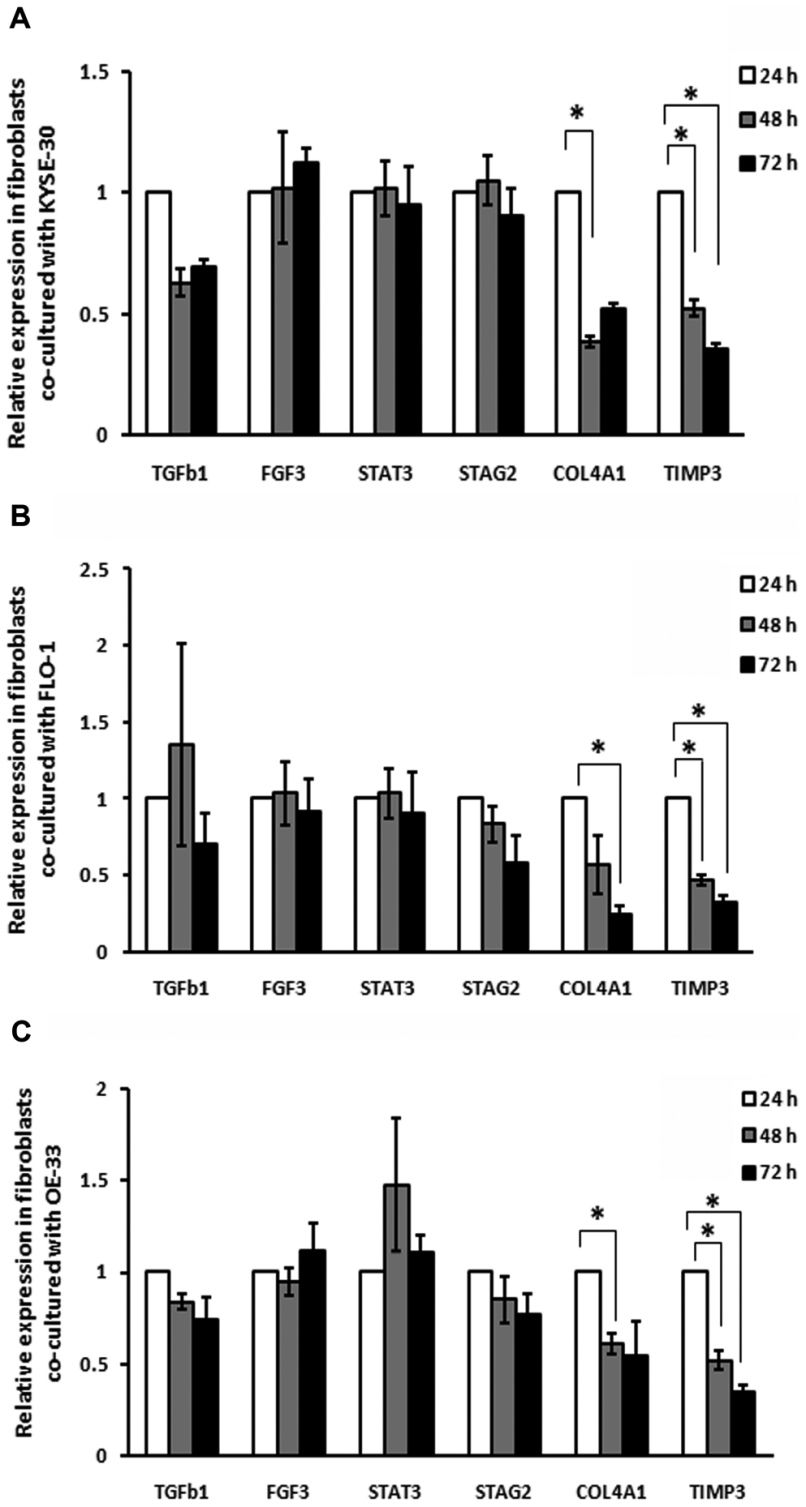
Expression analysis of 6 fibroblastic markers in normal fibroblasts co-cultured with esophageal cancer cell lines KYSE-30 (A), FLO-1 (B) and OE-33 (C). *COL4A1* expression in fibroblasts was significantly decreased after 2 days of incubation with KYSE-30 (P = 0.03) and OE-33 (P = 0.04) cells and after 3 days of incubation with FLO-1 cells (P = 0.01). *TIMP3* expression was *s*ignificantly decreased after 2 and 3 days of incubation with KYSE-30 (P = 0.0002 and P>0.0001, respectively), after 2 and 3 days of incubation with FLO-1 cells (P = 0.0013 and P = 0.0006, respectively) and after 2 and 3 days of incubation with OE-33 cells (P = 0.0017 and P = 0.0004, respectively) when compared to the measurement 24 h after the start of the co-culture.

We first selected 6 fibroblastic markers that were predicted by target prediction databases (Targetscan and/or DIANA-MicroT) to be potential targets for miR-21: *TGFβ1*, transforming growth factor beta1; *FGF1*, acidic fibroblast growth factor 1; *STAT3*, signal transducer and activator of transcription 3; *STAG2,* stromal antigen 2; *TIMP3*, tissue inhibitor metalloproteinase 3; and *COL4A1*, Collagen, type IV, alpha 1. We then analyzed the expression of these 6 fibroblastic markers in the normal fibroblast cell line HGF-1, as well as in HGF-1 cells co-cultured with KYSE-30, FLO-1 and OE-33, and found that HGF-1 normal fibroblasts expressed all 6 selected markers. Interestingly, *TIMP3* and *COL4A1* expression was reduced on the second and third day of incubation compared to the expression levels at the first day of co-culture (**Figure 5**). In addition, this reduction was negatively correlated with the expression of miR-21 in HGF-1 fibroblast cells co-cultured with any cell lines (KYSE-30, FLO-1 and OE-33) (**Figure 3** **; data not shown**). We then analyzed TIMP3 and COL4A1 protein expression in the HGF-1 – KYSE-30 and HGF-1 – FLO-1 co-culture systems and could not detect any TIMP3 protein, while COL4A1 was expressed, but the expression was not altered due to co-culture (**Figure S7**). The four other fibroblastic markers had similar and stable expression levels during the co-culture experiment, suggesting that their expression levels were not affected by the presence of cancer cells.

### Co-culture with cancer cells and expression of miR-21 induce the CAF marker S100A4 in HGF-1 fibroblasts

To investigate whether a CAF phenotype can be induced in normal fibroblasts, we co-cultured KYSE-30 cells and normal HGF-1 fibroblasts, and analyzed the expression of 4 CAF markers in HGF-1. Since signatures greatly vary depending on the tissue of origin [33]–[34] and since no ESCC-associated CAF markers have been identified so far, we selected 4 markers that have been found to be either ‘general’ CAF markers or oral/esophageal CAF markers [33], [35]–[37]: ACTA2 (or alpha-SMA), FAP, S100A4 (or FSP1) and CSPG4 (or NG2). Only S100A4 seems to be induced in HGF-1 fibroblasts both on RNA and protein level upon co-culture (**Figure S8A–B**). Moreover, up- or down-regulation of miR-21 in HGF-1 fibroblasts results in increased (**Figure S8C**) or decreased (**Figure S8D**) levels of S100A4, respectively. These results suggest that fibroblasts may be “activated” into a CAF phenotype, when residing in the proximity of cancer cells, and that miR-21 may be an important factor in this process.

## Discussion

In this study, we demonstrated that miR-21 is upregulated in esophageal tumors and that this upregulation is mainly confined to the cancer associated fibroblasts. MiR-21 overexpression has been reported in a variety of cancers including esophageal carcinoma (**Table 2**) [10], [13], [38]–[43], but only recently, scientists have started to study the miR-21 tissue localization pattern [20]–[21]. Previous studies in breast, lung and colon cancers have localized miR-21 mainly in the tumor stroma and more particularly in the stromal fibroblast-like cells. This localization may be due to factors secreted by cancer cells, which affect their microenvironment [20]–[21], [44]–[45]. On the contrary, Dillhof et al. and Qi et al. reported miR-21 expression in pancreatic and breast cancer cells but not in the surrounding stroma [8], [46]. Our *in situ* hybridization data detected miR-21 expression in fibroblast-like stromal cells adjacent to cancer cells. Accordingly, we detected significant miR-21 overexpression in FFPE tumor samples with high stromal content when compared to the samples with low stroma, further supporting the primary localization of miR-21 in the stromal components. Therefore, if more accurate data on miR-21 quantification in different tumor tissue samples is desired, the stromal content of the samples must be taking into account.

**Table 2:** MiR-21 overexpression in different histological types of esophageal cancer.

*Techniques*	*Pathology*	*Population*	*Findings*	*Statistical significance*	*Fold change*	*Ref.*
miRNA bioarray	ADC and SCC	American	Overexpression in Tumor vs. Normal	NA	5.2 (ADC to NE)	[13]
microarray	ADC and SCC	American/Canadian/ Japanese	Overexpression in Tumor vs. Normal	P<0.005	>2	[38]
qRT-PCR	SCC	Japanese	Overexpression in Tumor vs. Normal	P<0.0001	6.87	[10]
microarray	ADC	American	Overexpression in Tumor vs. Normal	P = 0.03	1.73	[39]
microarray	SCC	Japanese and cell line	Overexpression in Tumor vs. Normal; also in SCC cell line	P<0.05	NA	[40]
qRT-PCR	SCC	Japanese	Overexpression in Tumor vs. Normal	P<0.05	>2	[41]
microarray	SCC	Chinese	The most upregulated miRNA	P<0.05	24.2	[42]
qRT-PCR	SCC	Iran	Overexpression in Tumor vs. Normal	P = 0.0007	2.77	Present study

Abbreviations: ADC, adenocarcinoma; SCC, squamous cell carcinoma; NE, normal epithelium; NA, not available.

The tumor fibroblasts are “activated” normal cells that represent a modified state and are also termed peri-tumoral fibroblasts, reactive stromal fibroblasts or cancer associated fibroblasts (CAFs). CAFs facilitate the communication between tumor cells through cell-cell contact or paracrine/exocrine signaling, protease secretion and modulation of the extracellular matrix (ECM) [35], [47]. Yamamichi et al. and Yang et al. detected overexpression of miR-21 in CAFs from colorectal cancer and melanoma, respectively, and suggested that this deregulation could be caused by cancer-secreted cytokines [45], [47]. Nevertheless, the mechanisms of action of miR-21 in CAFs still remain unknown.

To unravel the contribution of miR-21 to the properties of the tumor stroma, we used a co-culture system for the KYSE-30 esophagus cell line and normal fibroblasts (HGF-1). Quantitative RT-PCR demonstrated miR-21 overexpression in both cell lines when co-cultured, but the impact of normal fibroblasts on KYSE-30 cells was clearly stronger. Moreover, the conditioned media without cells had a similar effect on KYSE-30 cells, which may highlight a role of normal fibroblasts in the tumor microenvironment by producing necessary factors that help promoting cancer progression and invasion. KYSE-30 cells that are co-cultured with normal fibroblasts show a higher propensity to migrate and invade through the matrix, which is a model for the ECM. Moreover, omitting the cells from this system and only incubating with the conditioned media from fibroblasts had similar effects on migration and invasion of cancer cells as the co-culture system. It has been demonstrated that miR-21 can be secreted within exosomes and can directly bind to Toll-like receptors, which are expressed on the cell surface of the immune cells, triggering an inflammatory response which leads to tumor growth and metastasis [48]. This may be important for the higher propensity of KYSE-30 cells to migrate and invade in our co-culture system.

By targeting distinct molecules such as matrix metalloproteinase regulators TIMP3 and TIAM1 [23], [25], miR-21 plays an essential role in cancer progression and metastasis.

In a co-culture system, we detected a time-dependent increase in miR-21 expression in the HGF-1 fibroblasts when co-cultured with the adenocarcinoma cell line FLO-1 while KYSE-30 cells showed a time-dependent increase of miR-21 expression when co-cultured with HGF-1 fibroblasts. This might be due to the inherent differences between *in vitro* and *in vivo* systems, which alter gene expression patterns. These patterns might be histology-dependent, and different histological types of cancer might have different levels of miR-21 expression.

We observed a downregulation of *TIMP3* and *COL4A1* in normal fibroblasts that were juxtaposed to esophageal cancer cells and this downregulation is consistent with the duration of co-culture and with the increase of miR-21 in the system. Both *TIMP3* and *COL4A1* are components of the ECM, and are validated as direct targets of miR-21 [15], [24], [49]–[51]. In this regard, we suggest that the vicinity of cancer cells can induce normal fibroblasts to become “active fibroblasts”, which produce higher levels of specific markers, including TIMP3 and COL4A1. With gradual induction of miR-21 in the system, these fibroblast markers are downregulated. However, as we cannot exclusively point out miR-21 as the only liable molecule for this downregulation, our observations need future refinement [23], [52].

Previously, TGFβ has been considered as the major regulator of the tumor microenvironment, thereby promoting tumor development and tumor growth [53]–[54]. TGFβ overexpression induces miR-21 processing, which in turn blocks apoptosis pathways and some tumor suppressor genes in cancer cells [26]. We detected miR-21 in the media of both cancer and normal cells, supporting a probable role of this miRNA as a signaling molecule in the tumor microenvironment.

S100A4, also known as fibroblast-specific protein 1 (FSP1), is a small acidic calcium-binding protein that transduces Ca(2+)-signals via interaction with intracellular target proteins [55] that has previously been described as a CAF marker associated with clinical outcome of cancer patients [56]. We detected higher levels of the CAF marker S100A4 in HGF-1 fibroblasts co-cultured with KYSE-30 or transfected with miR-21 precursor, and reduced expression when miR-21 was inhibited in HGF-1 fibroblast. This finding highlights the role of miR-21 in the induction of a CAF phenotype.

In conclusion we report that miR-21 is a microenvironment signaling molecule that contributes to tumor growth and cancer progression. Such data could lead to the development of new assays measuring miR-21 levels in tumoral stroma and/or body fluids for the prediction of SCC metastasis and survival.

## Supporting Information

Figure S1 **Primer validation for fibroblast specific marker genes.** Primer efficiencies were calculated according to the standard curves for each pair of primers: A) *TGFβ1*, B) *FGF1*, C) *STAT3*, D) *STAG2*, E) *TIMP3* and F) *COL4A1*. Quantitative RT-PCR was performed on serial concentrations of cDNA (2.00E-01, 4.00E-02, 8.00E-03, 1.60E-03, 3.20E-04 dilutions) and standard curves were analyzed with the BioRad CFX Manager software. All the calculated efficiencies were within the range of 90–110%.
(TIF)

Figure S2 **Selection of internal control genes for HGF-1 cells.** Eight different internal control genes were selected and qRT-PCR was performed on untreated HGF-1 cells, HGF-1 cells co-cultured with KYSE-30 (3 days of incubation) and HGF-1 cells co-cultured with OE-33 (3 days of incubation). Mean Cq value of each gene is shown in the graph. *HPRT1* had the lowest standard deviation (SD) but the Cq values were high. Therefore *β2M* was selected as the best normalizer gene with lowest SD, stable expression in all 3 analyzed samples and appropriate Cq values.
(TIF)

Figure S3 **In situ hybridization on FFPE tissue samples of 6 patients shows miR-21 upregulation in the stroma of the tumor but not in the stroma of adjacent normal squamous tissue.** The black arrows in the 40× magnification figure (right-bottom figure) show blue miR-21 signals in the cytoplasm of the fibroblasts.
(TIF)

Figure S4 **Basic expression of miR-21 in the FLO-1, OE-33, KYSE-30 and HGF-1 cell lines.** A) miR-21 was expressed in all analysed cell lines under normal conditions; data were normalized to U6 in each cell line; B) Mean Cq values for U6 expression in four analyzed cell lines. There is no significant difference in miR-21 expression between 4 cell lines.
(TIF)

Figure S5 **MiR-21 expression analysis in conditioned media obtained from HGF-1 and KYSE-30 cells.** Conditioned media of each cell line was collected from the same cell passage after 1, 2 and 3 days of incubation. All data were normalized to the 6 hour-old media which was set as time point 0. MiR-21 is significantly upregulated in conditioned media of normal HGF-1 fibroblast cells after 3 days of incubation (P = 0.02). In the conditioned media of KYSE-30 cells we observed higher miR-21 expression after 3 days of incubation, but this increase was not significant. P values were calculated with an unpaired *t* test with Welch' s correction.
(TIF)

Figure S6 **Migration and invasion assay for KYSE-30 cells co-cultured with HGF-1 fibroblasts in which miR-21 has been inhibited.** MiR-21 expression is significantly reduced in HGF-1 cells treated with miR-21 inhibitor during the migration (A) and invasion (B) assay. Cell migration (C) and invasion (D) properties of KYSE-30 seem to be reduced when co-cultured with HGF-1 cells in which miR-21 has been inhibited. *TIMP3* and *COL4A1* seem to be increased in HGF-1 cells treated with miR-21 inhibitor and co-cultured with KYSE-30 in the migration (E) and invasion (F) assay. *NC inh, negative control inhibitor; miR-21 inh, miR-21 inhibitor*.
(TIF)

Figure S7 **TIMP3 and COL4A1 protein expression in HGF-1 cells co-cultured with KYSE-30 (A) and FLO-1 (B).** No TIMP3 protein could be detected, while COL4A1 was expressed, but this expression was not significantly altered due to co-culturing with cancer cells.
(TIF)

Figure S8 **Expression of the CAF marker S100A4 in HGF-1 normal fibroblasts.** (A) Co-culture of HGF-1 with KYSE-30 induces *ACTA2*, *FAP* and *S100A4*, but not *CSPG4* gene expression. (B) Co-culture of HGF-1 with KYSE-30 induces S100A4, but not FAP, CSPG4 or ACTA2 protein expression. (C) Overexpression of miR-21 in HGF-1 cells leads to induction of S100A4, but not of ACTA2, FAP and CSPG4 protein expression. (D) Downregulating miR-21 in HGF-1 cells reduces S100A4, but not ACTA2, FAP and CSPG4 protein expression. *NC, negative control; inh, inhibitor*.
(TIF)

Methods S1 (DOC)

Table S1 **Gene specific primers used for qRT-PCR quantification of fibroblastic markers.**
(DOCX)

Table S2 **LinRegPCR analysis results.**
(DOCX)
