# Supplementary figures and images for: Expression, Tissue Distribution and Function of miR-21 in Esophageal Squamous Cell Carcinoma
Source: PLoS One. 2013 Sep 10;8(9):e73009. doi: 10.1371/journal.pone.0073009 (PMC3769386; doi:10.1371/journal.pone.0073009)

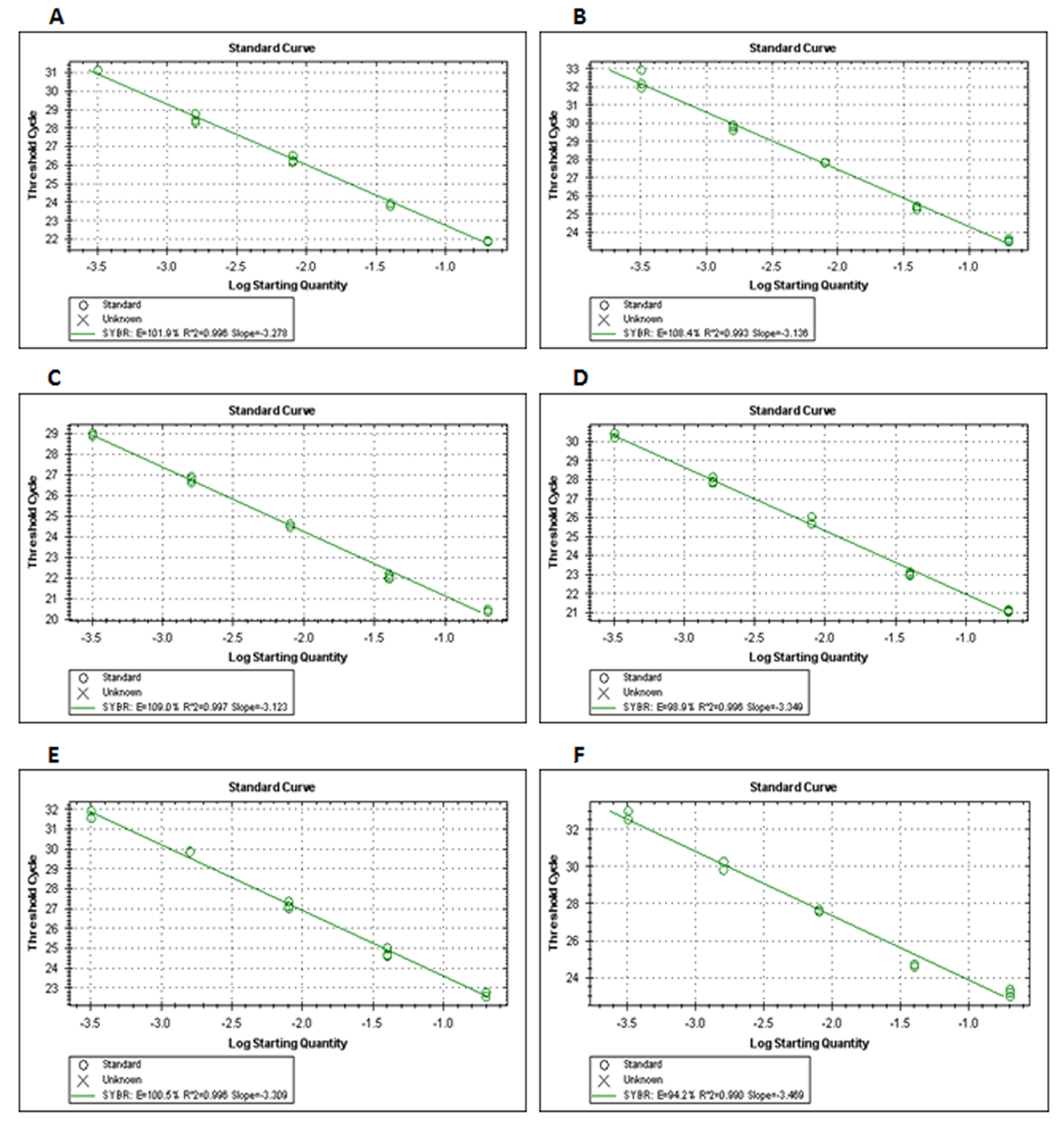

Supplement: Figure S1 — Primer validation for fibroblast specific marker genes. Primer efficiencies were calculated according to the standard curves for each pair of primers: A) TGFβ1, B) FGF1, C) STAT3, D) STAG2, E) TIMP3 and F) COL4A1. Quantitative RT-PCR was performed on serial concentrations of cDNA (2.00E-01, 4.00E-02, 8.00E-03, 1.60E-03, 3.20E-04 dilutions) and standard curves were analyzed with the BioRad CFX Manager software. All the calculated efficiencies were within the range of 90–110%. (TIF) [file pone.0073009.s001.tif]

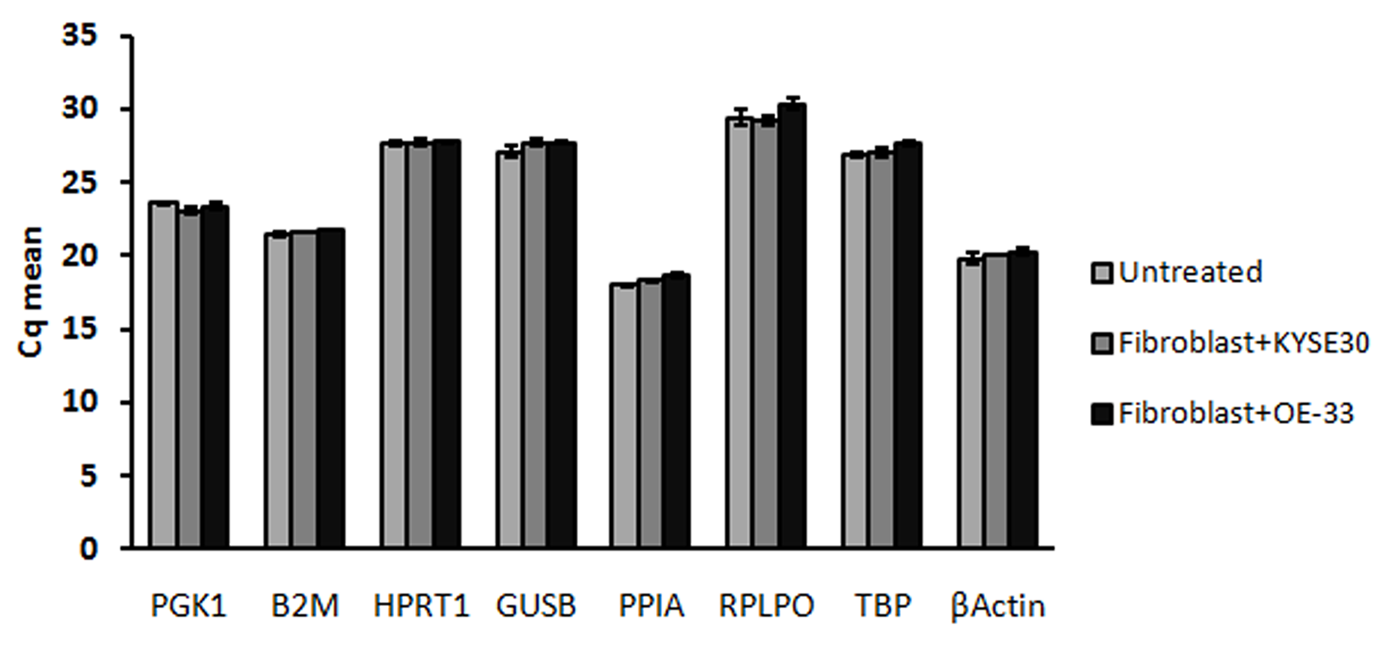

Supplement: Figure S2 — Selection of internal control genes for HGF-1 cells. Eight different internal control genes were selected and qRT-PCR was performed on untreated HGF-1 cells, HGF-1 cells co-cultured with KYSE-30 (3 days of incubation) and HGF-1 cells co-cultured with OE-33 (3 days of incubation). Mean Cq value of each gene is shown in the graph. HPRT1 had the lowest standard deviation (SD) but the Cq values were high. Therefore β2M was selected as the best normalizer gene with lowest SD, stable expression in all 3 analyzed samples and appropriate Cq values. (TIF) [file pone.0073009.s002.tif]

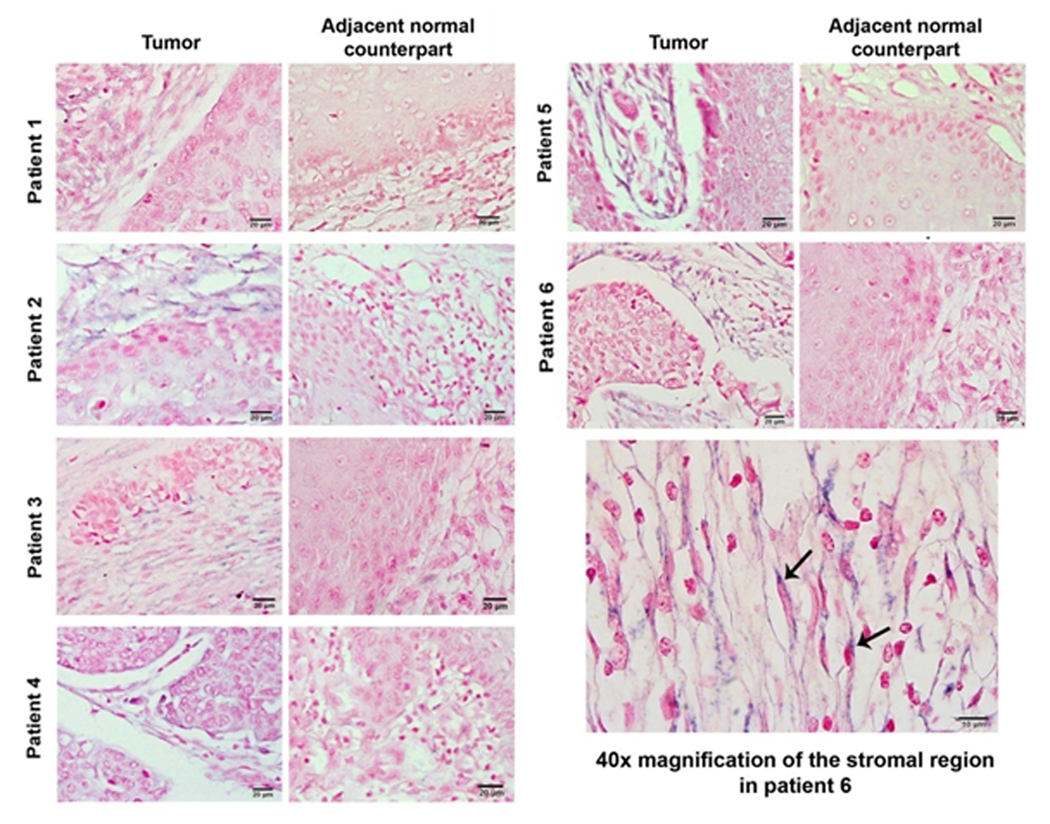

Supplement: Figure S3 — In situ hybridization on FFPE tissue samples of 6 patients shows miR-21 upregulation in the stroma of the tumor but not in the stroma of adjacent normal squamous tissue. The black arrows in the 40× magnification figure (right-bottom figure) show blue miR-21 signals in the cytoplasm of the fibroblasts. (TIF) [file pone.0073009.s003.tif]

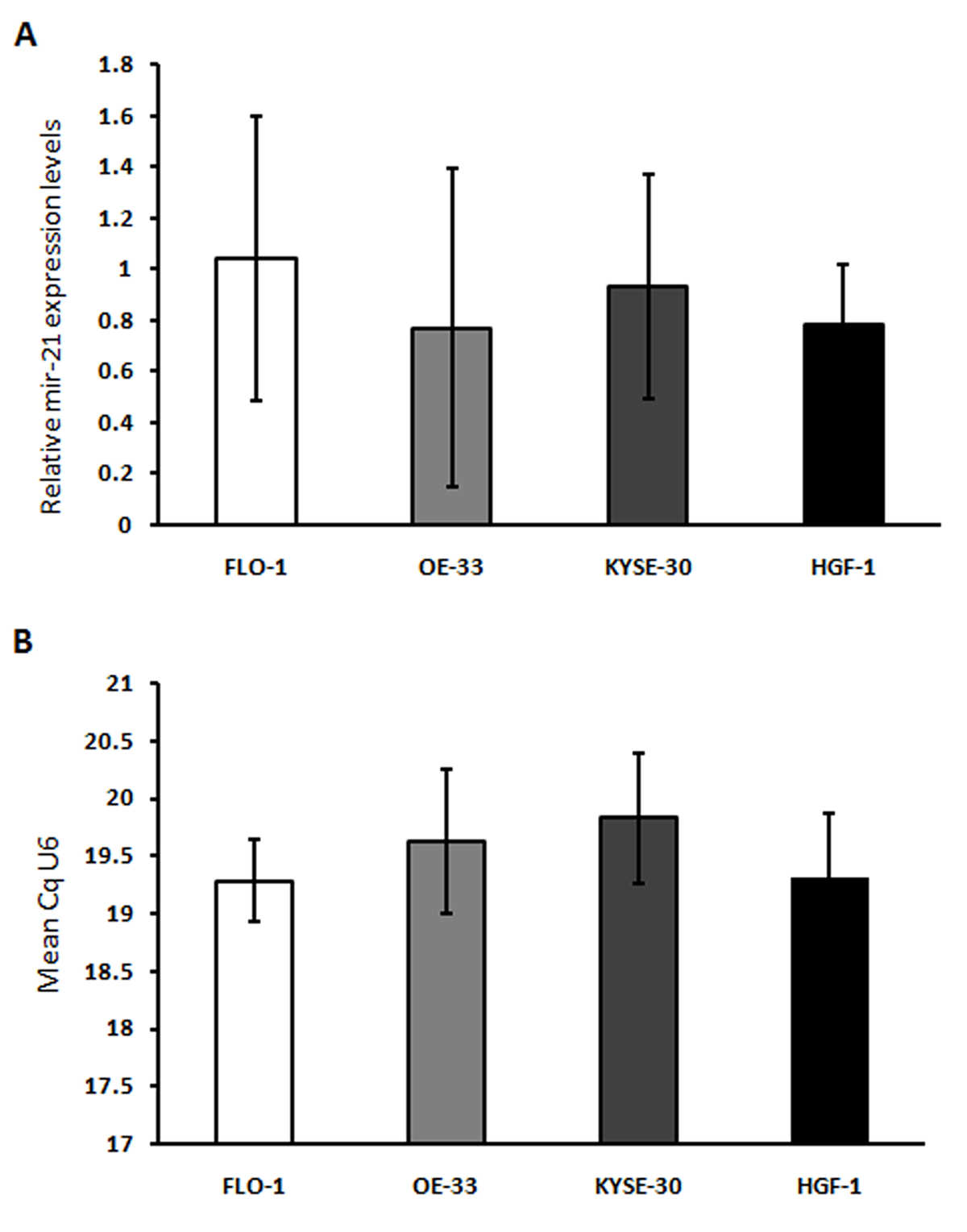

Supplement: Figure S4 — Basic expression of miR-21 in the FLO-1, OE-33, KYSE-30 and HGF-1 cell lines. A) miR-21 was expressed in all analysed cell lines under normal conditions; data were normalized to U6 in each cell line; B) Mean Cq values for U6 expression in four analyzed cell lines. There is no significant difference in miR-21 expression between 4 cell lines. (TIF) [file pone.0073009.s004.tif]

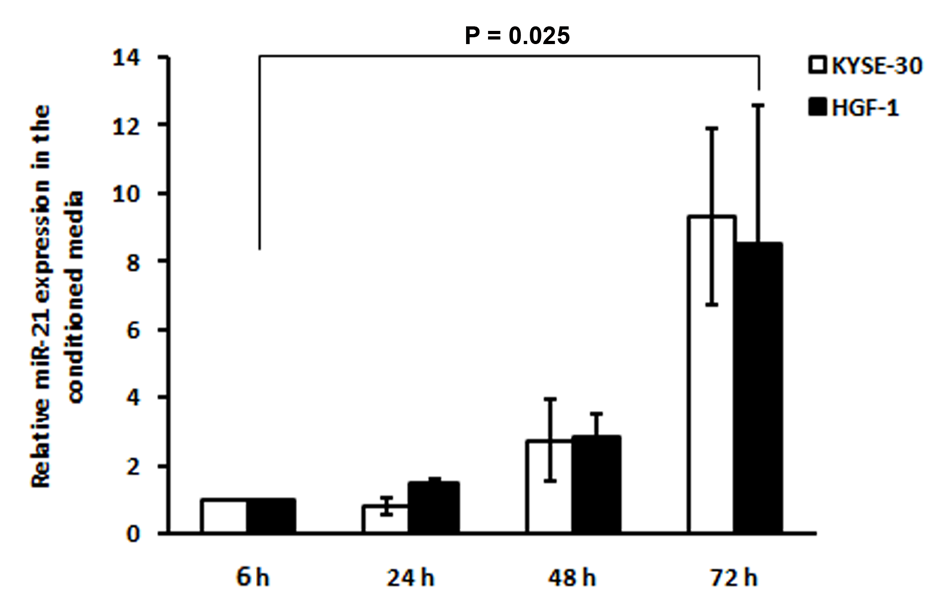

Supplement: Figure S5 — MiR-21 expression analysis in conditioned media obtained from HGF-1 and KYSE-30 cells. Conditioned media of each cell line was collected from the same cell passage after 1, 2 and 3 days of incubation. All data were normalized to the 6 hour-old media which was set as time point 0. MiR-21 is significantly upregulated in conditioned media of normal HGF-1 fibroblast cells after 3 days of incubation (P = 0.02). In the conditioned media of KYSE-30 cells we observed higher miR-21 expression after 3 days of incubation, but this increase was not significant. P values were calculated with an unpaired t test with Welch' s correction. (TIF) [file pone.0073009.s005.tif]

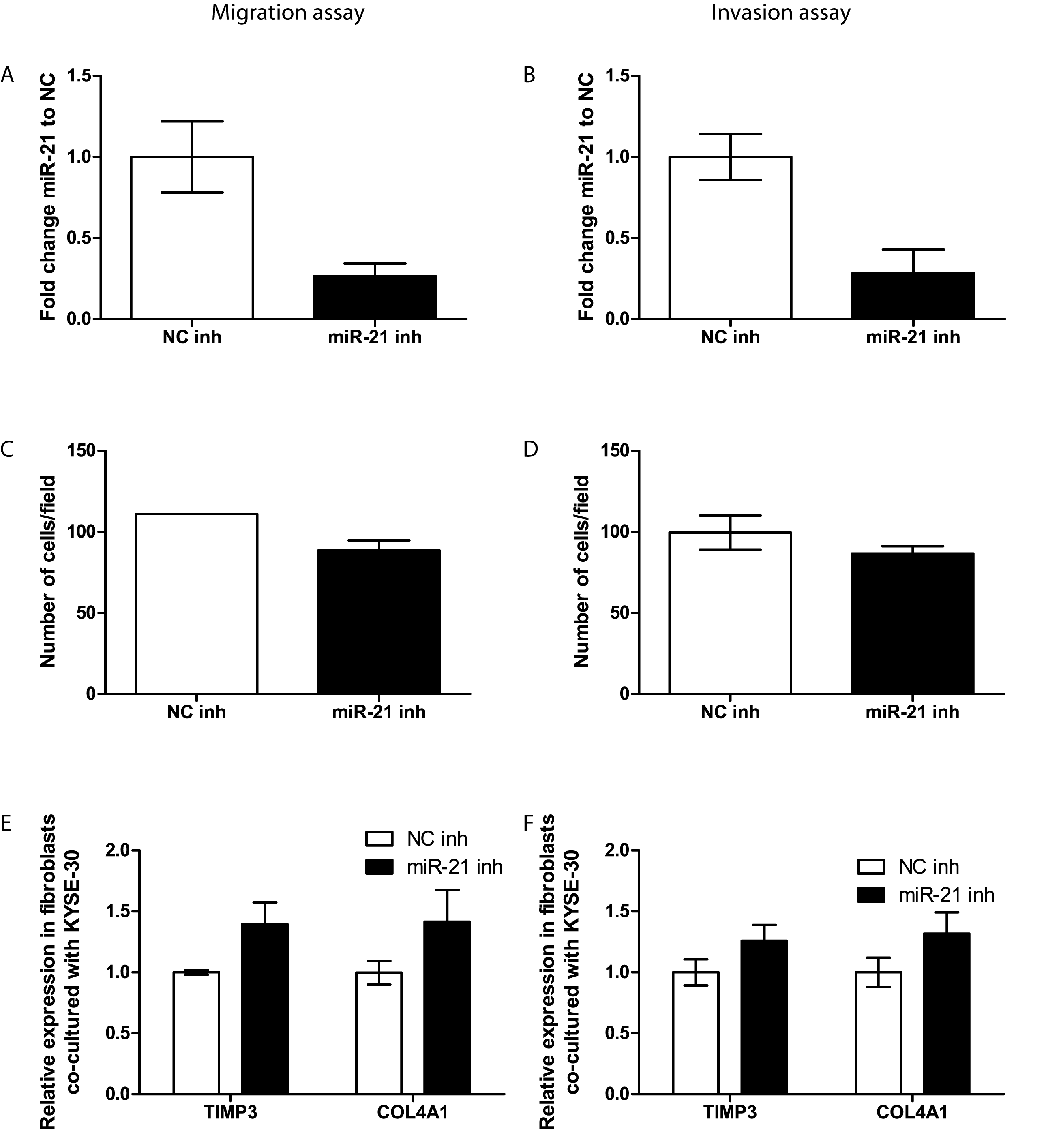

Supplement: Figure S6 — Migration and invasion assay for KYSE-30 cells co-cultured with HGF-1 fibroblasts in which miR-21 has been inhibited. MiR-21 expression is significantly reduced in HGF-1 cells treated with miR-21 inhibitor during the migration (A) and invasion (B) assay. Cell migration (C) and invasion (D) properties of KYSE-30 seem to be reduced when co-cultured with HGF-1 cells in which miR-21 has been inhibited. TIMP3 and COL4A1 seem to be increased in HGF-1 cells treated with miR-21 inhibitor and co-cultured with KYSE-30 in the migration (E) and invasion (F) assay. NC inh, negative control inhibitor; miR-21 inh, miR-21 inhibitor. (TIF) [file pone.0073009.s006.tif]

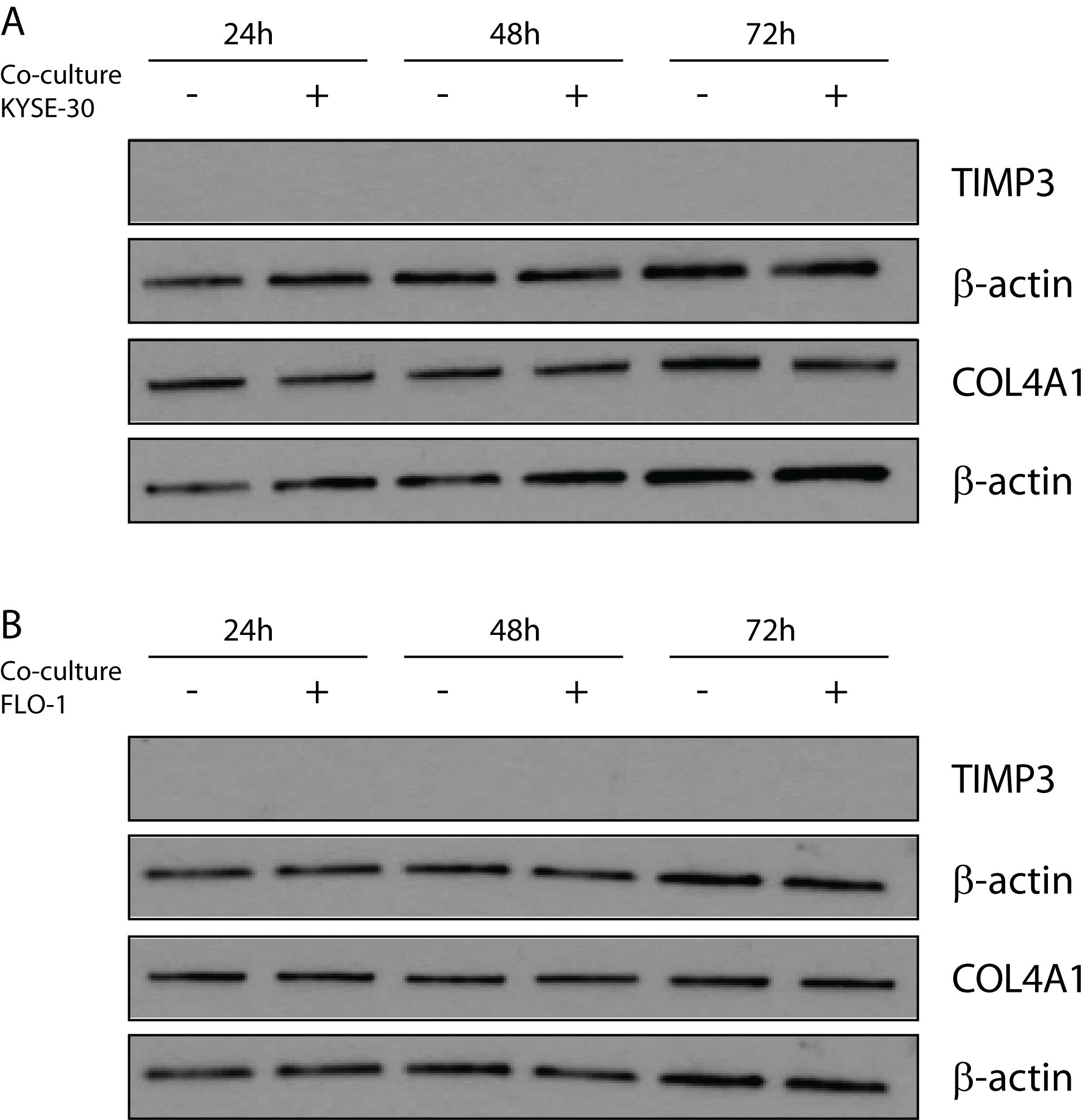

Supplement: Figure S7 — TIMP3 and COL4A1 protein expression in HGF-1 cells co-cultured with KYSE-30 (A) and FLO-1 (B). No TIMP3 protein could be detected, while COL4A1 was expressed, but this expression was not significantly altered due to co-culturing with cancer cells. (TIF) [file pone.0073009.s007.tif]

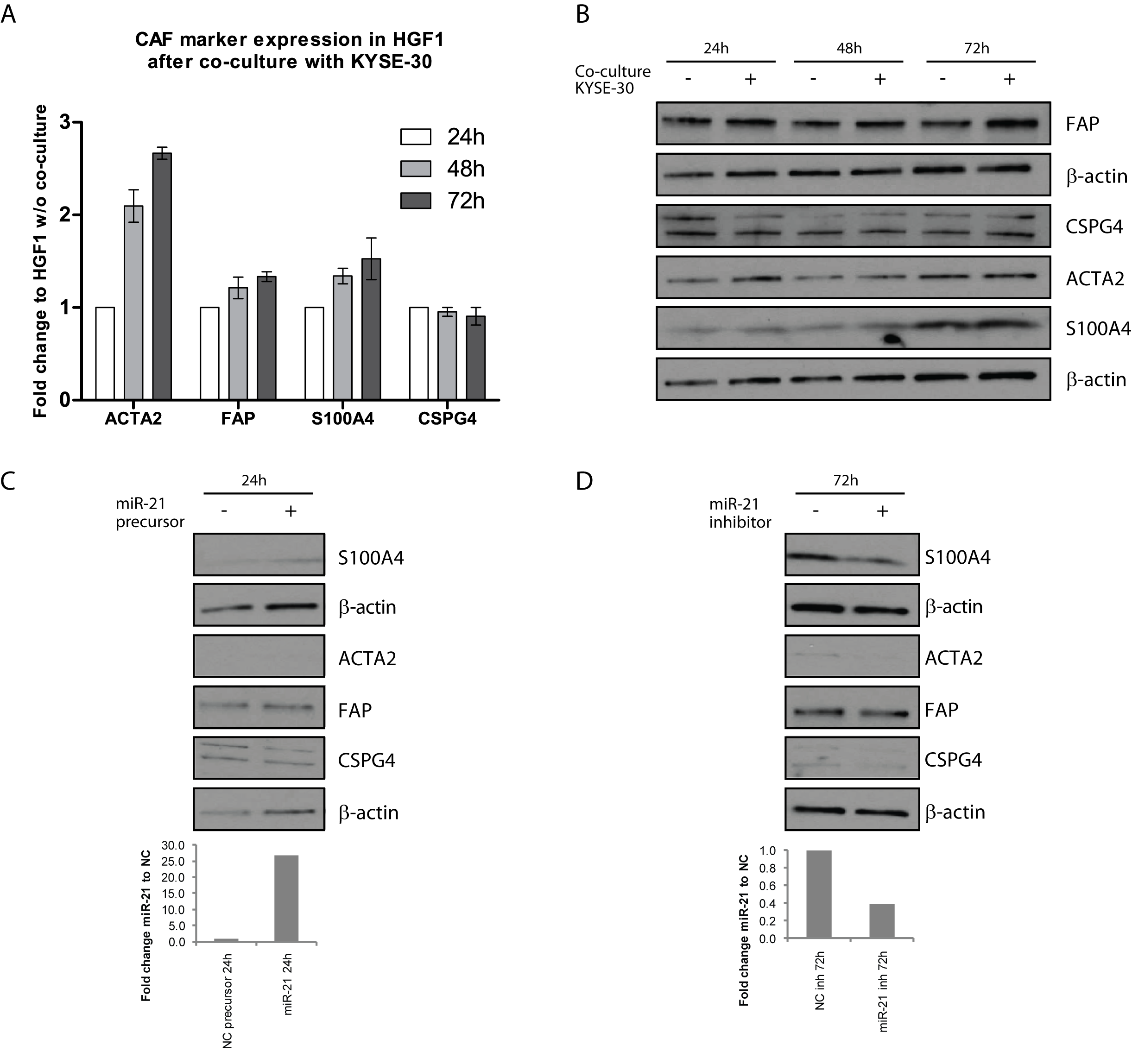

Supplement: Figure S8 — Expression of the CAF marker S100A4 in HGF-1 normal fibroblasts. (A) Co-culture of HGF-1 with KYSE-30 induces ACTA2, FAP and S100A4, but not CSPG4 gene expression. (B) Co-culture of HGF-1 with KYSE-30 induces S100A4, but not FAP, CSPG4 or ACTA2 protein expression. (C) Overexpression of miR-21 in HGF-1 cells leads to induction of S100A4, but not of ACTA2, FAP and CSPG4 protein expression. (D) Downregulating miR-21 in HGF-1 cells reduces S100A4, but not ACTA2, FAP and CSPG4 protein expression. NC, negative control; inh, inhibitor. (TIF) [file pone.0073009.s008.tif]
