# Supplementary material for: Expression, Tissue Distribution and Function of miR-21 in Esophageal Squamous Cell Carcinoma
Source: PLoS One. 2013 Sep 10;8(9):e73009. doi: 10.1371/journal.pone.0073009 (PMC3769386; doi:10.1371/journal.pone.0073009)
Supplement: Methods S1 — (DOC) [file pone.0073009.s009.doc]

SUPPORTING INFORMATION

Expression, tissue distribution and function of miR-21 in esophageal squamous cell carcinoma

Nazila Nouraee^1,2^, Katrien Van Roosbroeck^2^, Mohammad Vasei^3^, Shahriar Semnani^4^, Nader Mansour Samaei^5^, Farshad Naghshvar^6^, Abbas Ali Omidi^7^, George A. Calin^2*^, Seyed Javad Mowla^1*^

METHODS S1

### miR-21 upregulation and inhibition in HGF-1 cells

### To inhibit miR-21 in HGF-1 fibroblasts, we transfected HGF-1 cells with hsa-miR-21 Anti-miR miRNA Inhibitor or with Anti-miR Negative Control #1 (Life Technologies, USA) using Lipofectamine 2000 (Life Technologies, USA) according to the manufacturer’s instructions. Overexpression of miR-21 was achieved by transfection with hsa-miR-21-5 Pre-miR miRNA Precursor or with Pre-miR Negative Control 1 using Lipofectamine 2000 (Life Technologies) according to the manufacturer’s instructions.

Western blotting

Standard Western blotting procedures were used to analyze total cell lysates with the following antibodies: anti-TIMP3, anti-alpha smooth muscle actin (ACTA2), anti-fibroblast activation protein, alpha (FAP) (Abcam, UK), anti-collagen, type IV, alpha 1 (COL4A1), anti-fibroblast-specific protein (FSP1/S100A4) (LSBio, USA), anti-chondroitin sulfate proteoglycan 4 (CSPG4,NG2) (Proteintech, USA) and anti-actin (Sigma-Aldrich, USA).
