# Supplementary material for: Expression, Tissue Distribution and Function of miR-21 in Esophageal Squamous Cell Carcinoma
Source: PLoS One. 2013 Sep 10;8(9):e73009. doi: 10.1371/journal.pone.0073009 (PMC3769386; doi:10.1371/journal.pone.0073009)
Supplement: Table S1 — Gene specific primers used for qRT-PCR quantification of fibroblastic markers. (DOCX) [file pone.0073009.s010.docx]

Table S1. Gene specific primers used for qRT-PCR quantification of fibroblastic markers

| Gene | Accession number | Sequence | Length | Tm (⁰C) | Product size (bp) |
| --- | --- | --- | --- | --- | --- |
| *TGFβ1* | MIM:190180 | F: 5’-GAGCCTGAGGCCGACTACTA-3’ | 20 | 59.6 | 213 |
|  |  | R: 5’-CACGTGCTGCTCCACTTTTA-3’ | 20 | 60.0 |  |
| *FGF-1* | MIM:131220 | F: 5’-TGCCTCCAGGGAATTACAAG-3’ | 20 | 60.1 | 214 |
|  |  | R: 5’-TATAAAAGCCCGTCGGTGTC-3’ | 20 | 60.0 |  |
| *STAT3* | MIM:102582 | F: 5’-TTTGTCAGCGATGGAGTACG-3’ | 20 | 59.9 | 194 |
|  |  | R: 5’-GCTGCAACTCCTCCAGTTTC-3’ | 20 | 60.0 |  |
| *STAG2* | MIM:399826 | F: 5’-GCCACCATTTTACCCACGTA-3’ | 20 | 60.6 | 245 |
|  |  | R: 5’-ATTTCCACCTTTGCCCTTTT-3’ | 20 | 59.8 |  |
| *TIMP3* | MIM:188826 | F: 5’-GTGGGGAAGAAGCTGGTAAAG-3’ | 21 | 60.1 | 150 |
|  |  | R: 5’-CTCCAGCTTAAGGCCACAGA-3’ | 20 | 60.5 |  |
| *COL4A1* | MIM:120130 | F: 5’-TCCCTGGTGAAAGAGGAGAA-3’ | 20 | 59.8 | 152 |
|  |  | R: 5’-GGGCTGACATTCCACAATTC-3’ | 20 | 60.3 |  |
| *β2M* | MIM: 109700 | F: 5’-CACCCCCACTGAAAAAGATGAG-3’ | 22 | 63.0 | 106 |
|  |  | F: 5’-CCTCCATGATGCTGCTTACATG-3’ | 22 | 62.8 |  |
