# Supplementary material for: Expression, Tissue Distribution and Function of miR-21 in Esophageal Squamous Cell Carcinoma
Source: PLoS One. 2013 Sep 10;8(9):e73009. doi: 10.1371/journal.pone.0073009 (PMC3769386; doi:10.1371/journal.pone.0073009)
Supplement: Table S2 — LinRegPCR analysis results. (DOCX) [file pone.0073009.s011.docx]

Table S2. LinRegPCR analysis results

| Average | Experiment 7 | Experiment 6 | Experiment 5 | Experiment 4 | Experiment 3 | Experiment 2 | Experiment 1 |  |
| --- | --- | --- | --- | --- | --- | --- | --- | --- |
| 2.045 | 2.008 | 1.978 | 2.077 | 1.934 | 2.071 | 2.138 | 2.115 | miR-21 |
| 2.04 | 2.031 | 2.02 | 2.104 | 1.936 | 2.078 | 2.086 | 2.09 | 5S rRNA |

The primer efficiencies for miR-21 and 5S rRNA were determined by LinRegPCR analysis of all qRT-PCR data in FFPE patient samples. In total, 7 experiments were performed and the average efficiency of all experiments (which was ~2) was considered in our qRT-PCR data analysis.
